# Oxidative Stress Biomarkers in Oncology: Biological Mechanisms, Clinical Utility, and Organizational Considerations

**DOI:** 10.3390/antiox15101341

**Published:** 2026-10-16

**Authors:** Marta Pawłowska, Celestyna Mila-Kierzenkowska, Marek Jóźwiak, Jarosław Nuszkiewicz

**Affiliations:** 1 Department of Medical Biology and Biochemistry, Faculty of Medicine, Ludwik Rydygier Collegium Medicum in Bydgoszcz, Nicolaus Copernicus University in Toruń, 24 Karłowicza Street, 85-092 Bydgoszcz, Poland; jnuszkiewicz@cm.umk.pl; 2 Department of Organizational Innovation Management, Faculty of Management, Bydgoszcz University of Science and Technology, 7 Professor S. Kaliski Avenue, 85-796 Bydgoszcz, Poland; marek.jozwiak@pbs.edu.pl

**Keywords:** oxidative stress biomarkers, redox imbalance, cancer biology, clinical utility, precision oncology, biomarker implementation, quality of care, organizational considerations

## Abstract

Oxidative stress is a fundamental driver of carcinogenesis and tumor progression, arising from an imbalance between reactive oxygen or nitrogen species and antioxidant defense mechanisms. This redox disruption contributes to genomic instability, metabolic reprogramming, inflammation, immune modulation, and therapy resistance processes that together influence the biological behavior of cancer. A broad spectrum of oxidative stress biomarkers has been evaluated in oncology, including markers of lipid peroxidation, protein oxidation, DNA oxidation, and enzymatic or non-enzymatic antioxidant systems. This review integrates current mechanistic knowledge of the formation and functional significance of these biomarkers and evaluates clinical evidence regarding their diagnostic, prognostic, and predictive usefulness across multiple cancer types. We examine whether oxidative stress biomarkers could complement established measures of risk and describe treatment-related changes in systemic oxidative status, while distinguishing these potential applications from validated clinical uses. Despite promising findings, biomarker implementation remains limited by analytical variability, lack of standardized reference ranges, and insufficient prospective validation. Beyond biomedical considerations, the review also highlights key organizational and quality-related factors that may influence the integration of oxidative stress biomarkers into clinical practice. These include laboratory quality assurance requirements, methodological standardization, workflow and data management needs, and broader health system implications. By integrating mechanistic, clinical, analytical, and organizational evidence, this review identifies the validation and quality requirements that must be met before oxidative stress biomarkers can inform routine oncology decisions.

## 1. Introduction

### 1.1. Oxidative Stress as a Central Mechanism in Cancer Biology

Oxidative stress (OS) is one of the key mechanisms underlying cancer initiation and progression. It is defined as an imbalance between the production of reactive oxygen species (ROS) and reactive nitrogen species (RNS) and the antioxidant systems’ ability to neutralize them [1]. Under physiological conditions, ROS function as signaling molecules that regulate proliferation, differentiation, and the immune response [2]. However, chronic or excessive accumulation damages biological macromolecules, including lipids, proteins, and DNA, promoting mutagenesis and genomic instability [3,4].

In cancer cells, OS is particularly pronounced. Mitochondrial dysfunction, activation of NADPH oxidase (NOX), chronic inflammation, hypoxia-reoxygenation cycles, and oncogene activity increase ROS production [1,5,6]. Oxidative DNA damage, including nitrogenous base modifications and strand breaks, can initiate or accelerate neoplastic transformation [4,6]. At the same time, OS modulates signaling pathways associated with cell proliferation, apoptosis resistance, angiogenesis, and epithelial–mesenchymal transition, thereby supporting the development of an invasive phenotype [3,4].

Concurrently, cancer cells can adapt to a pro-oxidant environment by increasing antioxidant activity, enabling them to survive under high redox stress. This dynamic balance between ROS generation and buffering mechanisms is a crucial element of cancer biology and influences treatment response, particularly to therapies that employ pro-oxidant mechanisms, such as radiotherapy or selected chemotherapy regimens [3,4].

### 1.2. Oxidative Stress Biomarkers: Categories and Clinical Relevance

Excessive ROS activity forms stable oxidative damage products that can be measured in various biological materials. OS biomarkers encompass several main classes, reflecting damage to specific cellular structures and the effectiveness of the organism’s defense systems [7,8].

Lipid peroxidation markers, such as malondialdehyde (MDA), 4-hydroxynonenal (4-HNE), and oxidized low-density lipoprotein fractions, are formed as a result of free radical degradation of polyunsaturated fatty acids (PUFAs) in cell membranes. Their presence indicates membrane destabilization and the formation of reactive secondary aldehydes that can further modify proteins and DNA [8,9]. Protein oxidation markers, including carbonyl groups and advanced protein oxidation products, reflect permanent structural modifications of protein molecules, leading to impaired enzymatic and signaling functions [10]. Markers of oxidative DNA damage, particularly 8-oxo-7,8-dihydro-2′-deoxyguanosine (8-oxo-dG), are particularly important in oncology, as they indicate mutagenic changes in genetic material [11].

This assessment is complemented by parameters of antioxidant systems, including enzyme activities such as superoxide dismutase (SOD), glutathione peroxidase (GPx), and catalase (CAT), as well as indicators of total antioxidant capacity and the reduced-to-oxidized glutathione ratio [12]. These parameters assess the body’s ability to buffer OS and maintain redox homeostasis.

Growing interest in OS biomarkers in oncology stems from their potential to reflect dynamic biological processes not fully captured by genetic or histopathological analyses. They can provide information about the body’s current metabolic and inflammatory state, as well as the systemic oxidative burden associated with cancer [13].

### 1.3. Purpose and Scope of the Review

This paper aims to provide a comprehensive analysis of OS biomarkers in oncology, encompassing both the biological foundations and the clinical and organizational implications. First, the paper presents the molecular and biochemical basis for the development of key OS biomarkers, accounting for their sources in cancer cells and their roles in disease pathogenesis. Second, it reviews current clinical data on the diagnostic, prognostic, and predictive value of these biomarkers across various cancer types, with particular emphasis on observational and prospective studies. Third, it discusses the translational potential of these markers in precision medicine, including their potential applications in risk stratification, personalized therapy, and monitoring disease dynamics. Fourth, it presents organizational and qualitative issues related to implementing OS biomarkers in clinical practice, including standardizing analytical methods, ensuring quality assurance, and assessing healthcare systems’ readiness. This broad approach integrates biological, clinical, and systems perspectives, which is essential for responsibly introducing new diagnostic tools in oncology.

While numerous reviews have addressed the biological mechanisms of oxidative stress in cancer or the clinical associations of individual redox biomarkers, few have systematically integrated molecular, clinical, translational, and organizational dimensions within a single framework. The present review seeks to bridge these levels by linking mechanistic insights and clinical evidence with implementation science, laboratory quality governance, and health-system readiness considerations. By situating oxidative stress biomarkers within both precision oncology and healthcare delivery contexts, this work aims to clarify not only their biological relevance but also the practical conditions required for their responsible clinical adoption.

## 2. Materials and Methods

This review presents a narrative synthesis of literature on oxidative stress biomarkers in oncology, integrating biological, clinical, translational, and organizational perspectives. We identified relevant publications by searching PubMed/MEDLINE, Scopus, and Web of Science, supplemented by examining reference lists from relevant reviews and primary studies. Search terms concerned oxidative stress, individual biomarkers, cancer, clinical utility, and implementation of biomarker testing; examples include “oxidative stress”, “malondialdehyde”, “8-oxodG”, “protein carbonyls”, “cancer”, “prognostic”, and “standardization”. We did not retain the original search histories, so we cannot reproduce the exact database-specific queries.

We considered original clinical studies, including cross-sectional, case–control, cohort, and prospective investigations, as well as systematic reviews and meta-analyses relevant to oxidative stress biomarkers in oncology. We considered publications on analytical methods, clinical translation, and healthcare implementation when they addressed the review’s broader aims. We used mechanistic studies to explain biological plausibility, but distinguished their findings from evidence of clinical performance in humans. Publications focused exclusively on non-oncological models or antioxidant supplementation without measurement of oxidative stress biomarkers were outside the scope of this review.

We examined titles and abstracts for relevance to the review’s scope and consulted full texts when needed to assess their contribution to the narrative synthesis. We did not retain a record-level screening log. Therefore, we cannot reliably report the numbers of records identified, screened, excluded, and included, and we cannot reconstruct a study-selection flow diagram. We did not perform a meta-analysis. Instead, we synthesized the literature narratively, considering the consistency of findings across tumor types, the methods used to measure biomarkers, and the clinical relevance of reported outcomes.

In interpreting the clinical evidence, we gave greater weight to studies with clearly described analytical methods, reported sample sizes, multivariable adjustment for confounders, and clinically interpretable outcomes. We considered age, sex, smoking, body mass index, systemic inflammation, relevant comorbidities, diet, and anticancer treatment when interpreting comparisons. We retained studies without adequate adjustment for these factors where informative but treated their findings as descriptive associations and did not use them alone to support claims of tumor specificity or clinical utility. We used results from cell and animal models to explain biological plausibility and did not count them as evidence of performance in patients. For this narrative synthesis, we distinguished four types of clinical evidence: diagnostic discrimination, prognosis, treatment response prediction, and treatment monitoring. We described evidence as relatively stronger when findings were replicated in independent human cohorts, used clearly specified matrices and assays, addressed major confounders, and reported clinically interpretable outcomes or performance measures. We considered evidence moderate when associations were replicated, but analytical comparability, adjustment, or external validation remained incomplete. We considered evidence from a single small, unadjusted, or cross-sectional study limited. These descriptors are qualitative judgments for this review and do not constitute a formal certainty-of-evidence assessment.

When comparing clinical studies, we considered cancer type, study design, biological matrix, analytical method, sample size, the direction of biomarker changes relative to controls, and reported associations with clinical outcomes. We also considered whether analyses accounted for factors that can affect oxidative stress biomarkers, particularly age, smoking, body mass index, inflammation, comorbidities, and treatment exposure. We organized findings around biological mechanisms, clinical evidence, potential translational applications, and requirements for reliable laboratory implementation.

This narrative review does not provide pooled effect estimates or a formal risk-of-bias assessment. Because we did not retain the search histories and record-level screening log, we cannot fully reproduce the search and selection process or exclude selection bias. Differences in study design, biological matrices, analytical methods, and reporting units also limit direct comparison. Residual confounding and selective reporting may affect the apparent consistency of findings. Accordingly, the qualitative evidence descriptors used in this review should not be interpreted as formal ratings of study quality or certainty of evidence. Microsoft PowerPoint (version 2408; Microsoft Corporation, Redmond, WA, USA) was used for the final preparation of the figures.

## 3. Biological and Molecular Basis of Oxidative Stress Biomarkers

### 3.1. Biological Sources of Oxidative Stress in Tumors

One primary endogenous source of ROS in cancer cells is mitochondrial dysfunction, arising from impaired electron transport chain function and abnormal regulation of oxidative phosphorylation [14]. Another key microenvironmental source of OS in cancer is chronic inflammation. Tumor-infiltrating immune cells, particularly neutrophils and macrophages, produce significant amounts of ROS and RNS [15]. This is primarily associated with the activation of enzymes such as inducible nitric oxide synthase (iNOS), myeloperoxidase (MPO), NOX, and xanthine oxidase (XO) [16]. Another characteristic feature of the tumor microenvironment is cycles of hypoxia and reoxygenation, resulting from chaotic vascular architecture and unstable blood flow [17]. Hypoxic conditions disrupt mitochondrial function and cause reduced electron carriers to accumulate in upstream complexes (especially complexes I and III) by inhibiting cytochrome c oxidase (complex IV). In contrast, the rapid restoration of oxygen supply increases ROS production, e.g., via NOX and XO activation [18]. An additional intracellular source of ROS is deregulation of oncogenes and loss of tumor suppressor function. Activation of oncogenic drivers such as RAS, MYC, and BCR-ABL can alter cellular ROS production [19,20,21]. SIRT3 has context-dependent effects on mitochondrial metabolism and antioxidant defenses and cannot be characterized uniformly as a source of increased ROS. At the same time, inactivation of tumor suppressors such as p53, fumarate hydratase (FH), and succinate dehydrogenase (SDH) significantly alters cellular energy metabolism and promotes excessive ROS generation [22].

### 3.2. Formation and Biochemical Interpretation of Oxidative Stress Biomarkers

During cancer development, elevated ROS levels drive lipid peroxidation [19]. This process results from several interacting sources of oxidation reactions in cell membrane phospholipids, particularly those containing PUFAs, which are especially susceptible to oxidation. Oxidation of membrane lipids forms lipid peroxides, which are then degraded into reactive aldehydes such as 4-HNE and MDA [8]. These markers of increased lipid peroxidation have been demonstrated in various tumor types, including breast cancer [23]. Enzymes such as ACSL4 and LPCAT3 increase the incorporation of PUFAs into membrane phospholipids, which is an important element of the mechanism of so-called ferroptosis—an iron-dependent form of cell death characterized by excessive lipid peroxidation [24]. During cancer development, alongside lipid damage, oxidative protein modification occurs, including oxidation of amino acid residues, carbonyl group formation, tyrosine nitration, and formation of advanced oxidation protein products (AOPPs) [25]. The accumulation of oxidized proteins is a critical factor in cancer biology, driven by high metabolic activity, rapid proliferation, and increased ROS production [26].

**Figure 1:**
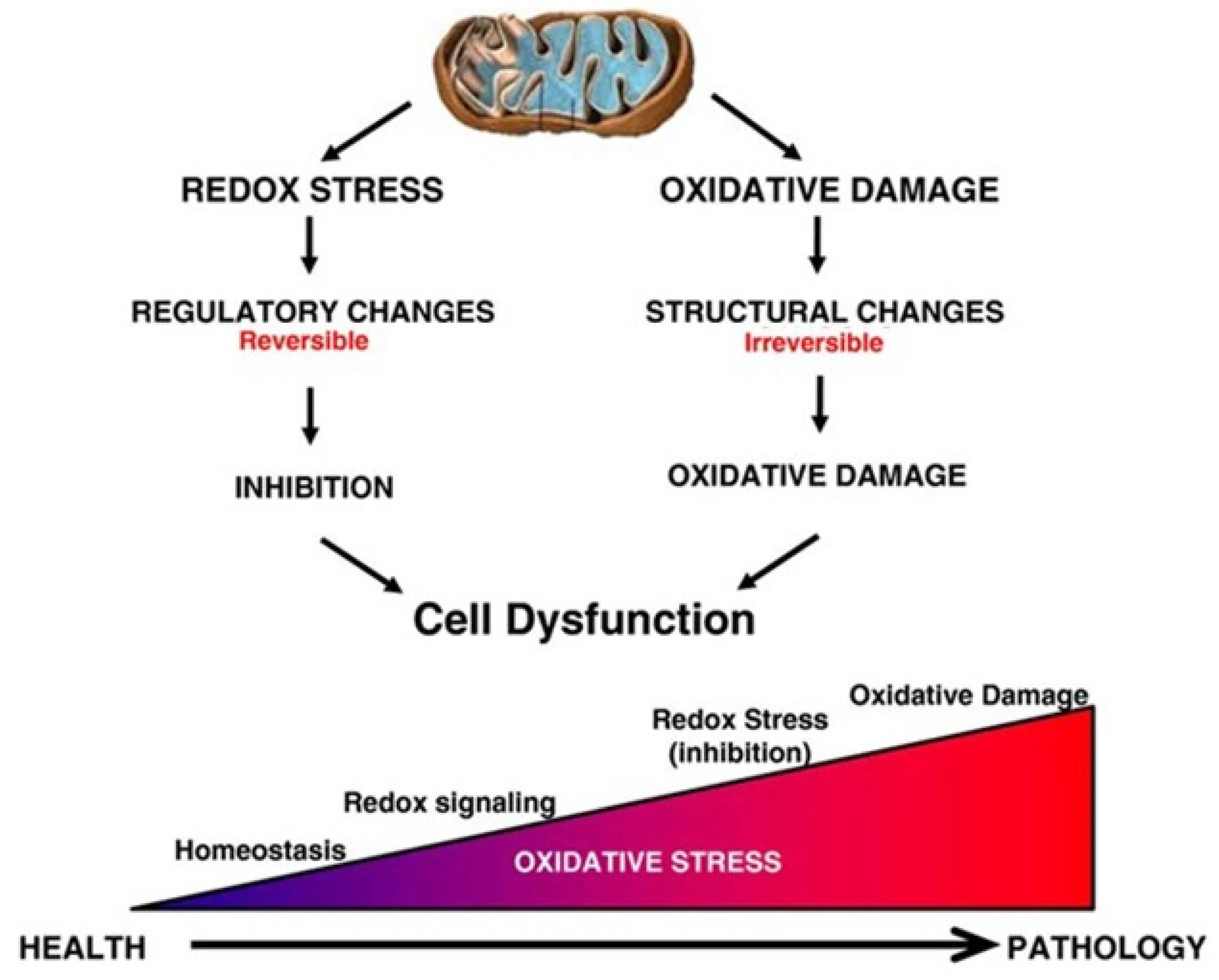
Conceptual framework of oxidative stress progression from physiological redox signaling to irreversible oxidative damage and cellular dysfunction.

Moreover, a specific aspect of OS in oncogenesis is damage to genetic material. The most characteristic effect of ROS reacting directly with DNA bases, which may lead to tumor formation, is 8-oxodG production. Accumulation of oxidative DNA lesions leads to genomic instability, point mutations, and chromosomal rearrangements, key mechanisms in neoplasm initiation and progression [1]. Oxidative DNA lesions can stall replication and activate the mammalian DNA damage response. Translesion synthesis may permit replication across some lesions, but can introduce mutations when lesion bypass is inaccurate. As a consequence, this shift increases the likelihood of mutagenic events and contributes to carcinogenic mutation [27]. Interpreting OS biomarkers also requires considering antioxidant systems, which help maintain redox signaling while limiting oxidative damage. In cancer cells, these systems are reprogrammed to enable OS tolerance, which promotes proliferation while preventing cell death [11]. The transcription factor nuclear factor erythroid 2–related factor 2 (NRF2) regulates antioxidant defense. Under basal conditions, KEAP1 binds NRF2 and facilitates its ubiquitination by a CUL3-based E3 ubiquitin ligase complex, targeting NRF2 for proteasomal degradation [4]. Under oxidative stress, reduced KEAP1-mediated degradation allows NRF2 to accumulate, translocate to the nucleus, and activate the transcription of antioxidant response element (ARE)-regulated genes [4]. These genes include TrxR1, SOD, GPx, and CAT [28]. As shown in Figure 1, excessive ROS production shifts redox balance from reversible signaling modifications toward structural macromolecular damage.

Under physiological conditions, redox signaling regulates cellular homeostasis through reversible post-translational modifications (e.g., S-glutathionylation, S-nitrosylation). Excessive reactive oxygen species production shifts the balance toward structural oxidative damage, including protein carbonylation and lipid peroxidation, ultimately contributing to cellular dysfunction and pathological processes.

### 3.3. Functional Consequences of Oxidative Stress in Cancer

OS biomarkers have been mechanistically linked to key stages of cancer development, and an expanding body of research indicates that ROS play critical roles in tumor initiation, progression, and resistance to therapy.

In tumor development, ROS can exert effects through epigenetic mechanisms [27]. Elevated levels of biomarkers reflecting an imbalance between ROS production and lipid peroxidation, such as 8-OHdG and MDA, are linked to early neoplastic alterations and mutation accumulation within cells [13]. Oxidative damage plays an oncogenic role by contributing to proto-oncogene activation and tumor suppressor gene inactivation, and by signaling abnormal cell growth and metastasis [29]. The key feature of epigenetic regulation in living organisms is DNA methylation, regulated by DNA methyltransferases (DNMTs) [30]. During the initiation phase, ROS-induced DNA damage, such as 8-OHdG formation, can perturb DNMT substrate recognition by interfering with DNA’s ability to serve as a substrate for this enzyme, potentially promoting malignancy [31]. OS can also induce chromosomal aberrations and translocations by affecting the mitotic spindle and endpoint checkpoints [32]. Moreover, elevated ROS can activate oncogenic signaling pathways, e.g., NF-κB (nuclear factor kappa B) and MAPK (mitogen-activated protein kinase), that drive cell proliferation and survival, linking oxidative stress signatures to early carcinogenic processes [31].

NF-κB generally promotes cell survival by inducing the expression of anti-apoptotic genes, including members of the Bcl-2 family and inhibitors of apoptosis proteins (IAPs) [33]. The MAPK/extracellular signal-regulated kinase (ERK) pathway, modulated by ROS, increases cellular proliferation [28]. A key growth-factor-responsive signaling cascade that is frequently hyperactivated in cancer is the phosphoinositide 3-kinase (PI3K) pathway. One of the principal ROS targets in this pathway is the inactivation of phosphatase and tensin homolog deleted on chromosome ten (PTEN), which leads to increased cyclin expression and accelerated progression through the cell cycle [34].

During tumor progression, chronically elevated ROS may create a dysfunctional tumor microenvironment (TME), leading to genomic instability by regulating redox-sensitive transcription factors and metabolic reprogramming, and contributing to tumor growth, invasion, and resistance to therapies [35]. Additionally, ROS regulate matrix metalloproteinase (MMP) activity and the Wnt/β-catenin signaling pathway, promoting tumor cell invasion and migration [36]. Increased cancer cell plasticity and their ability to survive stressful circulatory conditions may promote distant metastasis. In the primary tumor, MMPs released into the stiff extracellular matrix ECM modulate cellular behavior by facilitating local dissemination, migration, and invasion. They also influence intracellular signaling pathways and promote epithelial-to-mesenchymal transition (EMT), thereby enabling cells to acquire migratory/invasive properties [37]. OS plays an important role in EMT induction [38]. ROS trigger EMT by inducing transcription factors such as SNAIL and by transforming fibroblasts into cancer-associated fibroblasts (CAFs), which then release growth factors and cytokines that enhance tumor growth and metastasis [39]. Moreover, elevated ROS levels within the TME suppress T-cell proliferation and impair their anti-tumor activity, while ROS generated by other cells contribute to T-cell hyporesponsiveness in cancer patients [40]. The TME also harbors cytotoxic CD8+ T-cells that often express co-inhibitory receptors, such as programmed death-1 (PD-1). High OS levels are thought to upregulate PD-L1 and promote T-cell exhaustion, thereby facilitating immune evasion by tumor cells [41].

Angiogenesis, invasion, and migration are the final stages of malignant transformation. ROS support tumor angiogenesis by enhancing the expression and activity of vascular endothelial growth factor (VEGF) and hypoxia-inducible factor 1α (HIF-1α), thereby enabling oxygen and nutrient supply [26]. ROS stabilize HIF-1α even under conditions of relative normoxia by inhibiting prolyl hydroxylases responsible for its degradation. Stabilized HIF-1α induces VEGF expression and genes involved in angiogenesis [41]. NOX1 plays a critical role in stimulating angiogenesis in the TME, as it significantly upregulates VEGF mRNA levels and markedly elevates VEGF receptor expression in tumor vascular cells [42]. This creates a vicious cycle: hypoxia → ROS → HIF-1α stabilization → angiogenesis and metabolic adaptation. This may enable rapid tumor growth despite oxygen deprivation.

### 3.4. Experimental Evidence from In Vitro and In Vivo Models

In vitro and animal studies provide mechanistic evidence that redox pathways can influence tumor initiation, progression, and treatment resistance. They do not establish the clinical performance of oxidative stress biomarkers in patients. In vitro experiments show that cancer cells adapt to OS by overexpressing antioxidant enzymes and activating NRF2, enabling survival and promoting chemotherapy resistance [43]. In animal models, NRF2 has been shown to suppress tumor growth in the early stages of malignancy. Conversely, in later stages of tumor development, when ROS levels are elevated, NRF2 appears to protect against ROS-induced cell death in various models, including human cancers [11]. Increased NRF2 activity may contribute to resistance to some anticancer treatments by strengthening cellular antioxidant defenses [44]. Modulating redox pathways may affect cellular sensitivity to treatment, as suggested by experimental models of drug resistance in glioma and liver cancer [45]. Additionally, in vivo studies indicate that OS above the tolerance threshold of cancer cells can activate apoptosis, autophagy, ferroptosis, or other forms of cell death, underpinning therapeutic strategies that target redox modulation [46,47].

## 4. Clinical Evidence for Oxidative Stress Biomarkers in Oncology

### 4.1. Circulating Oxidative Biomarkers in Cancer Patients

Researchers have extensively investigated circulating OS biomarkers in cancer patients as minimally invasive indicators of systemic redox imbalance [48]. Most clinical studies have focused on peripheral blood-derived matrices, including serum, plasma, whole blood, and erythrocytes, as well as urine samples, which allow repeated measurements and dynamic assessment across disease and treatment stages [13]. A smaller subset of clinical investigations has evaluated oxidative markers directly in tumor tissue. However, most available evidence concerns circulating biomarkers that may reflect the global oxidative burden rather than tumor-specific processes [7]. The most frequently reported markers include products of lipid peroxidation (e.g., MDA, 4-HNE, oxidized LDL), protein oxidation markers (advanced oxidation protein products, protein carbonyls), DNA oxidation products (notably 8-oxodG), and indicators of antioxidant defense systems such as SOD, GPx, CAT, TAC, and the glutathione redox couple (GSH/GSSG) [49].

Interpretation of circulating oxidative biomarkers in oncology requires careful consideration of methodological and clinical heterogeneity. Differences in analytical techniques, biological matrices, pre-analytical handling, and units of measurement may substantially influence reported concentrations and limit cross-study comparability [50]. Moreover, circulating oxidative markers are affected not only by tumor presence but also by disease stage, treatment exposure, smoking status, body mass index, age, comorbidities, and systemic inflammatory activity [51]. Consequently, rather than presenting isolated findings for individual cancer types, the following subsections synthesize available evidence across major malignancies, with particular emphasis on recurring clinical patterns, consistency of directionality, and the overall robustness of the supporting data. Circulating biomarkers reflect oxidative processes throughout the body and cannot, on their own, distinguish tumor oxidative activity from the host response to cancer or from unrelated conditions. Smoking, obesity, chronic inflammation, comorbidities, diet, and anticancer treatment may influence their concentrations independently of tumor biology. Consequently, an association between a circulating marker and cancer should not be interpreted as evidence that the marker is tumor-derived or specific to a particular malignancy. Studies assessing tumor specificity should account for these factors and, where possible, compare circulating measurements with tumor tissue findings and appropriate clinical control groups.

#### 4.1.1. Lipid Peroxidation Markers

Products of lipid peroxidation, particularly MDA, represent the most extensively investigated circulating markers of oxidative stress in clinical oncology. Elevated circulating MDA has been reported in several solid tumors, but the direction and magnitude of the association are not uniform across cancer types and studies.

In one of the earlier case–control studies, Gönenç et al. [52] demonstrated significantly higher plasma MDA concentrations in patients with breast and lung cancer compared with healthy individuals. Notably, MDA was quantified using high-performance liquid chromatography (HPLC), a method considered more specific than conventional thiobarbituric acid-based assays. Importantly, in patients with breast cancer, plasma MDA levels were not significantly correlated with age, body mass index, or menopausal status within the studied cohort, suggesting that increased lipid peroxidation was associated with the presence of malignancy independent of major demographic factors. These findings provide early clinical evidence of a link between elevated circulating lipid peroxidation and solid tumors.

More robust data are derived from larger cohorts. In a study including over 200 patients with non-small cell lung cancer (NSCLC), Peddireddy et al. [53] reported significantly higher plasma lipid peroxidation products (MDA equivalents) than in controls, with values increasing with more advanced disease stage. These findings support elevated systemic lipid peroxidation in NSCLC and suggest a potential association with disease burden. However, despite this stage-related trend, evidence linking circulating MDA to clinically meaningful prognostic endpoints, such as survival or treatment response, remains limited. Furthermore, stratified analyses indicated potential modulation by smoking status, underscoring the importance of accounting for clinical confounders when interpreting circulating oxidative markers.

Consistent results have also been reported in breast cancer. Gupta et al. [54] found significantly elevated serum MDA concentrations in breast cancer patients compared with healthy controls, along with alterations in lipid profile and reduced antioxidant parameters. Similarly, Kilic et al. [55] demonstrated increased serum MDA levels in surgically treated breast cancer patients before adjuvant therapy, alongside elevated advanced oxidation protein products and thioredoxin levels. Importantly, participants in this study were non-smokers and had not received prior anticancer therapy, minimizing external influences on redox parameters. Collectively, these investigations support the reproducibility of elevated circulating lipid peroxidation markers in breast malignancies.

Head and neck cancers provide additional evidence. In patients with laryngeal and hypopharyngeal cancers, Manjunath et al. [56] observed significantly higher serum MDA levels than controls, along with reduced ferric-reducing antioxidant power. However, they found no statistically significant differences in MDA concentrations between early and advanced tumor stages or between patients with and without nodal involvement, suggesting that while lipid peroxidation increases in affected individuals, it may not reliably discriminate disease severity in this setting. Karaman et al. [57] reported comparable findings in laryngeal squamous cell carcinoma, where serum MDA levels were significantly elevated relative to healthy controls and correlated positively with markers of oxidative DNA damage. Notably, MDA levels in this study were not significantly influenced by smoking or alcohol status, indicating that tumor-associated oxidative imbalance persisted beyond traditional risk exposures.

Beyond individual primary studies, Mohideen et al. [58] conducted a systematic review and meta-analysis of MDA levels in oral squamous cell carcinoma (OSCC) across multiple biological matrices. The analysis showed significantly higher MDA concentrations in plasma, serum, and saliva samples from OSCC patients than in controls. Interestingly, some studies reported reduced MDA levels in tumor tissue, highlighting a potential divergence between circulating and local oxidative processes. From a clinical perspective, these findings reinforce the observation that circulating MDA frequently reflects systemic OS in oral malignancies.

Evidence from renal cell carcinoma further extends these observations. In a case–control study of biopsy-confirmed renal cell carcinoma, Panaiyadiyan et al. [59] reported significantly higher lipid peroxidation levels in patients than in matched controls. Although the study primarily focused on trace element imbalance, the accompanying increase in circulating lipid peroxidation markers, together with reduced glutathione peroxidase activity, supports systemic redox disturbance in urological malignancies.

Taken together, these studies demonstrate a recurring clinical pattern of elevated circulating lipid peroxidation markers across breast, lung, head and neck, oral, and renal cancers. Nevertheless, several aspects warrant careful interpretation. First, methodological heterogeneity is substantial, with MDA quantified using either HPLC-based methods or spectrophotometric thiobarbituric acid-reactive substance assays, which may contribute to variability in absolute values across studies [52,53,54,55,56]. Second, differences in patient characteristics, tumor stage distribution, smoking exposure, and nutritional status may influence reported concentrations [53,56,57]. Third, although some studies suggest associations between MDA levels and disease stage [53], others fail to demonstrate stage-dependent variation [56], indicating that the prognostic relevance of circulating lipid peroxidation remains context-dependent. However, the direction of MDA changes varies across malignancies. Studies of gastric cancer [60] and colorectal cancer [61] summarized in Table 1 reported lower serum MDA in patients than in controls. In both studies, this finding occurred alongside increased markers of protein and DNA oxidation. This contrasts with the increases in circulating MDA reported in several other cancer types. Differences in tumor type, patient characteristics, biological matrix, and analytical method may explain these divergent findings, but the available studies do not establish which factor accounts for the discrepancy. Consequently, elevated MDA should not be presented as a universal feature of cancer or interpreted as tumor-specific without considering the study context.

Overall, several solid tumors have shown increased circulating lipid peroxidation markers, although the direction of MDA changes is not consistent across studies. However, heterogeneity in analytical methodology, study design, and patient selection underscores the need for standardized measurement protocols and larger prospective studies before lipid peroxidation markers such as MDA can be reliably incorporated into routine oncological assessment.

#### 4.1.2. Protein Oxidation Markers

Researchers have investigated circulating markers of protein oxidation in multiple malignancies as indicators of systemic oxidative damage to plasma proteins. The most frequently assessed clinical markers include AOPP, protein carbonyls, and, less commonly, 3-nitrotyrosine as a marker of protein nitration [7]. In contrast to lipid peroxidation products such as MDA, clinical evidence for protein oxidation markers is smaller and more methodologically heterogeneous. Nevertheless, several case–control and population-based studies provide insight into recurring clinical patterns across solid tumors.

Evidence from gastrointestinal malignancies indicates consistently elevated circulating protein oxidation products. In patients with gastric cancer, Ma et al. [60] reported significantly higher serum concentrations of protein carbonyls, AOPP, and 3-nitrotyrosine than in healthy controls, indicating oxidative and nitrosative protein modifications. These findings were observed in newly diagnosed, histologically confirmed cases and were accompanied by elevated DNA oxidation markers, supporting systemic oxidative damage affecting multiple macromolecular targets. Similarly, in colorectal cancer, Chang et al. [61] observed significantly higher serum concentrations of protein carbonyls and AOPP in patients with colorectal cancer compared with matched healthy controls. These alterations were accompanied by reduced antioxidant defenses, including decreased activities of superoxide dismutase, catalase, and glutathione peroxidase, indicating a systemic pro-oxidative profile in colorectal cancer patients.

More recent studies have confirmed these findings across additional tumor types. In a cross-sectional study including patients with breast and colon cancer, Kundaktepe et al. [62] demonstrated significantly elevated serum levels of protein carbonyls and AOPP in both malignancies compared with healthy individuals. Importantly, these changes were observed in newly diagnosed patients before systemic therapy and were associated with other markers of oxidative imbalance, including increased prooxidant-antioxidant balance and decreased ferric reducing antioxidant power, indicating a shift toward a systemic pro-oxidative state. These results support the hypothesis that oxidative modification of circulating proteins is a common feature of diverse solid tumors.

Head and neck malignancies provide additional evidence for altered protein oxidation. In patients with oral squamous cell carcinoma, Nayyar et al. [63] demonstrated significantly elevated serum AOPP concentrations in untreated, histologically confirmed cases compared with healthy controls. The study design excluded patients with systemic diseases or conditions affecting protein metabolism, strengthening the interpretation that elevated AOPP levels were directly associated with malignant disease. These findings indicate that oxidative modification of plasma proteins is present in patients with established oral squamous cell carcinoma and may reflect a systemic oxidative profile associated with malignant disease.

Beyond cross-sectional comparisons, epidemiological studies have examined associations between protein oxidation markers and breast cancer. In a population-based case–control study involving over 2000 participants, Rossner et al. [64] reported higher odds of breast cancer among women with higher plasma protein carbonyl concentrations. The association was strongest in the highest quartile (OR 1.6) and remained after adjustment for several measured risk factors. However, the case–control design does not establish that protein carbonyl levels predict future cancer development or that the association is independent of all potential confounders.

Zipprich et al. [65] also reported an association between plasma protein carbonyl concentrations and breast cancer status among sisters with a family history of the disease. The association was not strictly monotonic across concentration quartiles. These findings add to the evidence of an association in this specific population, but do not establish that protein carbonyls predict future breast cancer or contribute causally to its development.

However, associations between protein oxidation markers and cancer risk are not uniformly observed across malignancies. In a large nested case–control analysis within the Prostate Cancer Prevention Trial, involving over 3600 participants, Hoque et al. [66] found no significant association between baseline serum protein carbonyl levels and subsequent prostate cancer risk. Odds ratios across quartiles of protein carbonyl concentration did not differ significantly from unity, and no associations emerged when stratifying by tumor grade or treatment group. These findings indicate potential tumor-type specificity and suggest that circulating protein oxidation markers may not universally predict cancer risk across all malignancies.

Taken together, available clinical data indicate that circulating protein oxidation markers, particularly AOPP and protein carbonyls, are frequently elevated in patients with gastrointestinal, breast, and head and neck malignancies and, in some settings, correlate with increased disease risk. Observing these elevations before anticancer treatment reduces the likelihood that they result from therapy, but does not establish tumor specificity. Inflammation, comorbidities, and other host factors may also contribute.

Nonetheless, substantial heterogeneity persists across studies. Analytical methods vary considerably, including spectrophotometric assays based on dinitrophenylhydrazine derivatization for protein carbonyl detection and spectrophotometric or ELISA-based techniques for AOPP quantification, limiting direct comparability. Sample sizes range from relatively small clinical cohorts to large population-based investigations, and pre-analytical factors, inflammatory status, nutritional state, and comorbid conditions may influence circulating concentrations. Furthermore, although epidemiological evidence supports associations between protein oxidation markers and cancer risk in certain malignancies, prospective validation and standardized clinical cut-off values remain lacking.

Overall, protein oxidation markers demonstrate biologically plausible and clinically observable elevations in several tumor types, reflecting systemic oxidative imbalance and macromolecular damage. However, their integration into routine oncological risk assessment or clinical monitoring requires further methodological standardization, mechanistic clarification, and large-scale prospective validation.

#### 4.1.3. DNA Oxidation Markers

Markers of oxidative DNA damage, most commonly 8-hydroxy-2′-deoxyguanosine (8-OHdG) and 8-oxodG, have been extensively investigated in clinical oncology as circulating indicators of systemic OS [67]. These biomarkers are most frequently measured in urine, typically normalized to creatinine, or in serum and plasma, allowing minimally invasive assessment across different stages of disease and treatment [68].

Across multiple malignancies, elevated circulating or urinary levels of 8-OHdG/8-oxodG have been consistently reported in cancer patients compared with healthy controls. In a large case–control study including 222 patients with newly diagnosed, previously untreated non-small cell lung cancer (NSCLC) and 207 matched controls, Peddireddy et al. [53] demonstrated significantly higher urinary 8-oxodG excretion in cancer patients (6.06 ± 0.81 vs. 4.36 ± 0.69 ng/mg creatinine). Urinary 8-oxodG levels increased with advancing disease stage, with the most pronounced differences observed in stages III–IV. Multivariable analysis identified smoking status and advanced disease stage as the strongest predictors of elevated urinary 8-oxodG, while age and sex were not significant predictors. In addition, urinary 8-oxodG correlated positively with plasma lipid peroxidation products, indicating concurrent oxidative damage across different biomolecular compartments within the same patient population.

Similar findings have been reported in head and neck malignancies. Murugaiyan et al. [69] observed significantly increased urinary 8-OHdG levels in patients with newly diagnosed oral cancer compared with healthy controls. In contrast, patients evaluated 1 year after treatment exhibited significantly lower urinary 8-OHdG concentrations, approaching those observed in controls. These findings indicate that elevated oxidative DNA damage markers may be particularly evident in untreated disease and may decrease following clinical management.

Evidence from gastrointestinal malignancies further supports the presence of increased oxidative DNA damage in cancer patients. In gastric cancer, Ma et al. [60] reported significantly elevated serum 8-OHdG levels compared with healthy controls, along with increased markers of protein oxidation and nitration. Notably, lipid peroxidation markers in the same cohort showed different patterns, highlighting potential variability in oxidative stress profiles across biomolecular targets. Urinary measurements have shown consistent findings. Chen et al. [70] reported significantly higher urinary concentrations of both 8-OHdG and 8-hydroxyguanosine (8-OHG) in untreated patients with gastric cancer than in controls, with receiver operating characteristic analysis showing moderate discriminative performance (AUCs of 0.777 and 0.841, respectively).

In colorectal cancer (CRC), elevated urinary oxidative DNA damage has also been consistently documented. Skalska-Bugala et al. [71] reported significantly higher urinary 8-oxodG levels in patients with CRC compared with controls. At the same time, patients with inflammatory bowel disease also exhibited increased levels, indicating that elevated oxidative DNA damage may reflect both malignant and inflammatory conditions. Similarly, Acevedo-León et al. [72] reported significantly increased urinary 8-oxodG concentrations in CRC patients, along with alterations in antioxidant and prooxidant parameters. They also observed correlations between oxidative stress markers and tumor-related biomarkers, further supporting the link between systemic oxidative imbalance and malignancy.

Beyond circulating and urinary measurements, researchers have also assessed oxidative DNA damage in peripheral blood cells. Lagadu et al. [73] reported significantly higher levels of 8-oxodG in peripheral blood mononuclear cell DNA from patients with esophageal cancer compared with controls, with differences remaining significant after adjustment for demographic and technical factors. These findings suggest that elevated oxidative DNA damage may be detectable across multiple biological compartments in cancer patients.

While case–control studies consistently demonstrate elevated DNA oxidation markers in established malignancy, prospective evidence regarding their predictive value remains less consistent. In a large population-based cohort study including 8793 participants, Gao et al. [74] found no significant association between urinary oxidized guanine derivatives and subsequent risk of lung, colorectal, breast, or prostate cancer in the overall population. Although nominal associations were observed in selected subgroups, these did not remain significant after adjustment for multiple testing, indicating limited evidence for their role as independent predictors of future cancer incidence.

Despite consistent evidence of elevated oxidative DNA damage in patients with lung, oral, gastric, colorectal, and esophageal cancers, considerable variability exists across studies. Differences in biological matrices, analytical methods, patient characteristics, and clinical factors such as smoking status, inflammation, and treatment exposure may influence measured concentrations. These factors contribute to inter-study heterogeneity and complicate direct comparisons across clinical populations.

Overall, current clinical evidence supports a recurring pattern of elevated circulating and urinary markers of oxidative DNA damage across a wide range of malignancies. However, variability in study design and limited prospective validation indicate that, although DNA oxidation markers are promising indicators of systemic OS, they require further standardization and large-scale longitudinal investigation before routine integration into clinical oncology practice.

#### 4.1.4. Synthesis of Clinical Patterns

Collectively, clinical studies report alterations in circulating markers of lipid, protein, and DNA oxidation in patients with several malignancies, although the direction and magnitude of these changes vary across studies. MDA is elevated in several malignancies, but studies of gastric and colorectal cancer have also reported lower serum concentrations than in controls [60,61]. Its associations with tumor stage and clinical outcomes are likewise inconsistent [75]. Similarly, protein oxidation markers, including protein carbonyls and AOPP, are frequently increased in cancer patients and, in some populations, have been associated with increased disease risk or unfavorable clinical features [25].

Markers of oxidative DNA damage, most commonly urinary or circulating 8-OHdG/8-oxodG, also demonstrate reproducible elevation in several cancer types and, in some studies, correlate with disease stage or tumor-related biomarkers. However, prospective evidence suggests that their predictive value for future cancer risk in the general population remains limited [74].

Alongside increased oxidative damage markers, many studies report alterations in antioxidant defense systems, including reduced activities of superoxide dismutase, catalase, and glutathione peroxidase, further supporting a systemic redox imbalance [76]. Nevertheless, considerable heterogeneity exists across studies, reflecting differences in analytical methods, biological matrices, patient characteristics, and clinical confounders.

Overall, circulating OS biomarkers can reflect systemic oxidative alterations in cancer patients, but their clinical added value remains unclear. However, their interpretation requires careful consideration of methodological variability and disease-specific context. Table 1 summarizes representative clinical studies.

**Table 1:** Clinical studies evaluating circulating oxidative stress biomarkers in cancer patients.

Cancer Type	Biomarkers	Biological Matrix	Sample Size (Cases/Controls)	Direction vs. Controls	Key Clinical Findings	Reference
Breast cancer, lung cancer	MDA	Plasma	38/41	↑ MDA	Plasma MDA levels were significantly higher in cancer patients and were not influenced by age, BMI, or menopausal status	[52]
NSCLC	MDA, 8-oxodG, SOD, GPx	Plasma, urine, erythrocytes	222/207	↑ MDA, ↑ 8-oxodG, ↓ SOD, ↓ GPx	Urinary 8-oxodG and plasma MDA significantly higher in patients; levels increased with tumor stage; smoking and advanced stage were strongest predictors; oxidative markers inversely correlated with antioxidant enzyme activity	[53]
Breast cancer	MDA, TAC, SOD, GSH-Px	Serum	30/100	↑ MDA	Serum MDA significantly increased and antioxidant defenses reduced in untreated breast cancer patients compared with healthy controls	[54]
Breast cancer	MDA, AOPP, thioredoxin, SOD	Serum	23/13	↑ MDA, ↑ AOPP, ↑ thioredoxin, ↑ SOD	Significantly elevated oxidative stress markers and antioxidant enzyme activity in newly diagnosed, untreated, non-smoking breast cancer patients	[55]
Laryngeal and hypopharyngeal cancer	MDA, FRAP	Serum	50/40	↑ MDA, ↓ FRAP	Significantly elevated serum MDA and reduced antioxidant capacity (FRAP) in cancer patients; no association with tumor stage or nodal status	[56]
Laryngeal squamous cell carcinoma	MDA, 8-OHdG, PON1	Serum	29/21	↑ MDA, ↑ 8-OHdG, ↓ PON1	Serum MDA and 8-OHdG significantly higher and PON1 activity lower in cancer patients; positive correlation between MDA and 8-OHdG; oxidative imbalance independent of smoking and alcohol status; no significant association with tumor stage	[57]
Renal cell carcinoma	LPO, GSH-Px, SOD	Serum	76/64	↑ LPO, ↓ GSH-Px, ↔ SOD	Significantly increased lipid peroxidation and decreased glutathione peroxidase activity in RCC patients, indicating systemic oxidative imbalance; SOD levels were not significantly different	[59]
Gastric cancer	8-OHdG, protein carbonyls, AOPP, 3-nitrotyrosine, MDA	Serum	30/30	↑ 8-OHdG, ↑ protein oxidation markers, ↓ MDA	Increased oxidative DNA damage and protein oxidation, but decreased lipid peroxidation in gastric cancer patients, indicating an altered systemic oxidative stress profile	[60]
Colorectal cancer	Protein carbonyls, AOPP, 8-OHdG, SOD, CAT, GPx, MDA	Serum	36/40	↑ protein oxidation markers, ↑ 8-OHdG, ↓ antioxidants, ↓ MDA	Elevated protein and DNA oxidation with reduced antioxidant enzyme activity and decreased lipid peroxidation in colorectal cancer patients	[61]
Breast cancer, colon cancer	Protein carbonyls, AOPP, MDA, PAB, IMA, NOx, FRAP	Serum	90/35	↑ protein oxidation, ↑ MDA, ↑ prooxidant balance, ↓ FRAP	Multiple oxidative stress markers significantly elevated in newly diagnosed cancer patients; impaired antioxidant capacity; oxidative markers associated with tumor characteristics including metastasis, tumor size, and HER2 status	[62]
Oral squamous cell carcinoma	AOPP	Serum	30/25	↑ AOPP	Serum AOPP levels are significantly elevated in untreated OSCC patients, indicating increased systemic protein oxidation	[63]
Breast cancer risk (case–control)	Protein carbonyls	Plasma	1050/1107	↑ protein carbonyls associated with higher odds of breast cancer in this case–control study	Higher plasma protein carbonyl levels associated with significantly increased breast cancer risk; strongest association observed in highest quartile (OR 1.6, p-trend = 0.0001)	[64]
Breast cancer risk (sisters discordant for breast cancer)	Protein carbonyls	Plasma	266/326	↑ protein carbonyl levels associated with breast cancer status in this familial sample	Plasma protein carbonyl levels were associated with breast cancer status among sisters; the association was observed in intermediate and higher concentration quartiles and was not strictly monotonic.	[65]
Prostate cancer risk (nested case–control)	Protein carbonyls	Serum	1808/1805	No significant difference	Serum protein carbonyl levels not associated with prostate cancer risk or tumor grade in a large prospective cohort	[66]
Oral cancer	8-OHdG, MDA, FRAP	Urine, serum	30/30	↑ 8-OHdG, ↑ MDA, ↓ FRAP	Urinary 8-OHdG and serum MDA significantly elevated in oral cancer patients, particularly in newly diagnosed cases; oxidative markers decreased after treatment follow-up; inverse correlation between 8-OHdG and antioxidant capacity (FRAP)	[69]
Gastric cancer	8-OHdG, 8-OHG	Urine	60/70	↑ 8-OHdG, ↑ 8-OHG	Urinary 8-OHdG and 8-OHG significantly elevated in gastric cancer patients; both biomarkers demonstrated moderate diagnostic performance (AUC 0.777 and 0.841, respectively), indicating discrimination between the study groups, without establishing clinical diagnostic utility	[70]
Colorectal cancer	8-oxodG	Urine	121/45	↑ 8-oxodG	Urinary 8-oxodG significantly elevated in colorectal cancer patients compared with controls; elevated levels also observed in inflammatory bowel disease but not in adenoma, supporting its association with malignant and inflammatory conditions	[71]
Colorectal cancer	8-oxodG, CAT, GSH, GSSG	Urine, serum	80/60	↑ 8-oxodG, ↑ GSSG, ↓ CAT, ↓ GSH	Urinary 8-oxodG and prooxidant markers significantly increased, while antioxidant markers decreased in CRC patients; oxidative markers correlated with tumor markers (CEA, CA19-9) and inflammatory markers, and were more pronounced in advanced disease stages	[72]
Esophageal cancer	8-oxodG	PBMC DNA	17/43	↑ 8-oxodG	Significantly higher 8-oxodG levels in PBMC DNA of esophageal cancer patients compared with controls, indicating increased systemic oxidative DNA damage	[73]

Abbreviations used: 8-OHdG—8-hydroxy-2′-deoxyguanosine; 8-OHG—8-hydroxyguanosine; 8-oxodG—8-oxo-7,8-dihydro-2′-deoxyguanosine; AOPP—advanced oxidation protein products; AUC—area under the curve; BMI—body mass index; CA19-9—carbohydrate antigen 19-9; CAT—catalase; CEA—carcinoembryonic antigen; CRC—colorectal cancer; FRAP—ferric reducing antioxidant power; GPx—glutathione peroxidase; GSH—reduced glutathione; GSH-Px—glutathione peroxidase; GSSG—oxidized glutathione; HER2—human epidermal growth factor receptor 2; IMA—ischemia-modified albumin; LPO—lipid peroxidation; MDA—malondialdehyde; NOx—nitric oxide metabolites (nitrite/nitrate); NSCLC—non-small cell lung cancer; OR—odds ratio; OSCC—oral squamous cell carcinoma; PAB—prooxidant–antioxidant balance; PBMC—peripheral blood mononuclear cells; PON1—paraoxonase 1; RCC—renal cell carcinoma; SOD—superoxide dismutase; TAC—total antioxidant capacity. ↑, increased; ↓, decreased; ↔, no significant difference compared with controls.

### 4.2. Clinical Utility and Limitations of Oxidative Stress Biomarkers in Oncology

A difference between patients and healthy controls establishes an association, not diagnostic accuracy in the intended clinical population. Prognostic value requires an association with a future outcome independent of established prognostic factors. Predictive value requires evidence that biomarker status modifies the benefit of a specified treatment, whereas treatment-associated change requires longitudinal changes that improve a clinical decision. These endpoints should not be inferred from one another. Table 2 summarizes the biological materials, commonly used analytical methods, and pre-analytical limitations of selected oxidative stress biomarkers, together with their current status in oncology.

Researchers have extensively investigated circulating OS biomarkers for clinical utility in oncology, including applications in cancer detection, risk stratification, prognostic assessment, and treatment monitoring [77]. The cumulative clinical evidence indicates that multiple classes of oxidative biomarkers, including lipid peroxidation products, protein oxidation markers, indicators of oxidative DNA damage, and antioxidant parameters, are consistently altered in cancer patients compared with healthy individuals [78]. These findings show that systemic redox alterations can be measured in cancer patients, but they do not, by themselves, establish tumor specificity or clinical utility.

From a diagnostic perspective, OS biomarkers show reproducible differences between cancer patients and control populations, supporting their potential as adjunct indicators of disease [79]. Nevertheless, substantial overlap between patient and control groups limits their specificity and sensitivity, and current evidence does not support their use as standalone diagnostic tools. Their clinical value may therefore lie in combination with established tumor biomarkers or in multi-parameter biomarker panels rather than as independent diagnostic indicators [80].

Researchers have also evaluated OS biomarkers for prognostic and predictive applications. Associations between oxidative damage markers and indicators of disease burden, including tumor stage, tumor-related biomarkers, and systemic inflammatory activity, suggest that these parameters may reflect disease severity and tumor-related oxidative burden [81]. However, these relationships are not consistently observed across all malignancies, and prospective studies have not yet established their independent prognostic value. Similarly, epidemiological studies suggest that oxidative protein damage markers may be associated with increased cancer risk in certain populations, although these findings are not universally reproducible [82,83].

Longitudinal observations further indicate that OS biomarkers may reflect dynamic changes during cancer treatment. Reductions in oxidative damage markers following therapeutic intervention suggest partial restoration of systemic redox balance, supporting their potential utility as indicators of treatment-related oxidative responses. However, available longitudinal data remain limited, and the clinical significance of such changes requires further validation [84,85].

Several important limitations currently restrict the clinical implementation of OS biomarkers in oncology. These include substantial methodological heterogeneity, lack of standardized analytical protocols, variability in reporting units, and the influence of non-cancer-related factors such as age, smoking, comorbidities, and systemic inflammation. In addition, most available evidence comes from cross-sectional or case–control studies, with limited prospective validation and insufficiently defined clinical decision thresholds. Table 3 summarizes the current clinical readiness of the biomarkers discussed in this review, along with the evidence needed before they can inform clinical decisions.

Overall, OS biomarkers represent promising indicators of systemic redox alterations in cancer patients, with potential applications in risk assessment and treatment monitoring. However, significant methodological variability and insufficient prospective validation currently preclude their routine clinical use. Further standardized, large-scale longitudinal studies are required to clarify their diagnostic and prognostic value and to define their role within integrated biomarker frameworks in oncology.

## 5. Translational Applications of Oxidative Stress Biomarkers in Oncology

Translational medicine focuses on translating biological knowledge into clinical practice by identifying diagnostic and predictive tools that support therapeutic decision-making [86,87]. In oncology, OS biomarkers represent a promising, though still developing, area of such knowledge transfer. While previous research has shown their connection to tumor biology, determining how to use them systematically and usefully in clinical practice remains crucial [81,88]. The translational potential of redox biomarkers lies in their ability to reflect dynamic metabolic and adaptive processes that are not fully captured by classical models based solely on genetic or histopathological analysis [86].

### 5.1. Potential Roles in Clinical Decision-Making

Introducing OS biomarkers into oncology practice requires demonstrating their added value over current diagnostic tools. From a translational perspective, their ability to enhance risk stratification and support personalized therapy is particularly important [81].

One of the most promising translational applications of OS biomarkers lies in refined biological risk stratification [81]. Disturbances in redox balance reflect the cumulative impact of tumor metabolic activity, chronic inflammation, hypoxia–reoxygenation cycles, environmental exposures, and the patient’s intrinsic antioxidant capacity [7]. Clinical studies have reported changes in lipid peroxidation, oxidative DNA damage, and protein oxidation markers across several cancer types. However, the direction and consistency of these changes vary by biomarker, malignancy, and study [53,60,61,89]. In selected cohorts, higher levels of oxidative damage markers have been associated with more advanced disease stage or greater tumor burden, suggesting that systemic oxidative imbalance may reflect biological aggressiveness rather than merely the presence of malignancy [53,81]. Associations between circulating oxidative markers and TNM stage are inconsistent across studies. Whether a composite oxidative profile provides clinically useful information beyond established staging and prognostic factors remains untested and requires prospective validation [81,89].

A second translational dimension concerns treatment personalization [13]. Many standard oncologic therapies, including radiotherapy and several chemotherapeutic agents, exert cytotoxic effects by generating reactive oxygen species. The therapeutic window of such interventions depends, in part, on the redox balance within tumor cells and on their ability to activate antioxidant defense mechanisms [90,91]. Experimental evidence highlights the role of NRF2-driven antioxidant reprogramming in promoting resistance to oxidative injury [92], while excessive ROS accumulation beyond a tolerable threshold can induce apoptosis, autophagy, or ferroptosis [90]. Clinical observations further indicate that altered antioxidant enzyme activities often coexist with elevated oxidative damage markers in cancer patients, reflecting complex adaptive responses [13]. Baseline redox profiling may help investigate differences in oxidative damage and antioxidant responses between patients. However, current clinical evidence does not establish that circulating oxidative stress biomarkers identify tumor susceptibility to prooxidant therapy or improve treatment selection. Prospective studies should test their added value alongside genomic and clinical information [91,92].

Cancer metabolism, inflammation, treatment exposure, and host physiology can all influence circulating oxidative stress biomarkers. Reported correlations with tumor markers or inflammatory parameters suggest possible associations with disease activity, but they do not establish that these biomarkers measure the tumor’s metabolic burden. Serial measurements may describe changes in systemic oxidative status; whether such changes provide information beyond established clinical assessments requires prospective study [81,93,94,95].

The greatest clinical value of OS biomarkers is likely to emerge within integrated, multivariate frameworks rather than through isolated measurements. A composite redox signature incorporating markers of lipid peroxidation, protein oxidation, oxidative DNA damage, and antioxidant capacity may better capture the complexity of redox biology than any single parameter. Such an approach aligns with the broader movement toward multiparametric modeling in precision oncology, where functional, inflammatory, and metabolic indicators are combined with genomic data to generate more comprehensive patient profiles. Within this paradigm, OS biomarkers can be conceptualized as a functional layer that contextualizes molecular alterations within the tumor’s dynamic biochemical environment.

### 5.2. Integration into Precision Oncology Frameworks

Precision oncology has evolved around identifying actionable mutations, molecular subtypes, and pathway-specific vulnerabilities. Genomic and transcriptomic profiling have transformed clinical practice by enabling targeted therapies and improving prognostic classification [96,97]. Nevertheless, static mutational landscapes alone cannot fully explain cancer biology. Tumor progression and therapeutic response are shaped by dynamic processes, including inflammation, metabolic reprogramming, and redox regulation, which continuously interact with genetic alterations [98]. In this broader biological context, OS biomarkers add a functional dimension that complements molecular profiling and may deepen the interpretative depth of precision medicine [99,100].

Accumulating experimental and clinical evidence demonstrates that oxidative DNA damage, redox-sensitive signaling pathways, and antioxidant defense mechanisms are closely intertwined with genomic instability and mutagenesis [11,101]. ROS contribute to lesion formation, such as 8-oxodG, which may induce base mispairing and promote mutational events if not adequately repaired [102]. At the same time, tumors often show alterations in DNA repair pathways and redox-regulatory signaling systems, including dysregulation of the NRF2–KEAP1 axis, further shifting the balance between oxidative damage and repair [103]. Integrating oxidative biomarkers with genomic data may help investigate relationships between oxidative injury, DNA repair, and signaling pathways, but the clinical value of such combined profiles remains to be validated. Two tumors sharing similar driver mutations may differ substantially in their redox status, reflecting differences in mitochondrial function, microenvironmental hypoxia, inflammatory infiltration, or antioxidant adaptation. Such divergence may influence therapeutic susceptibility, particularly to treatments that exploit oxidative mechanisms, and may partially explain heterogeneity in clinical outcomes among genetically comparable cases [11].

Beyond the interaction between redox imbalance and DNA repair, OS operates at the intersection of inflammation and metabolism. Chronic inflammatory signaling promotes sustained ROS production, while metabolic reprogramming characteristic of malignant cells alters mitochondrial dynamics, glycolytic flux, and lipid composition, all of which modulate oxidative balance [31]. This interdependence supports an integrated inflammation–metabolism–OS axis that shapes tumor behavior and host response [1]. Combining redox biomarkers with inflammatory and metabolic parameters is a plausible approach to studying patient heterogeneity, but prospective validation is needed to determine whether it improves stratification beyond established clinical and molecular measures [72]. Such multidimensional profiling can reveal biologically coherent patterns, distinguishing tumors characterized by high inflammatory activity and oxidative burden from those dominated by alternative adaptive mechanisms [31,72].

The development of integrated “redox signatures” represents a logical extension of this approach [81,104]. Composite profiles combining markers of lipid peroxidation, protein oxidation, oxidative DNA damage, and antioxidant capacity warrant investigation, but it remains unclear whether they are more reliable or clinically informative than individual markers [105]. Clinical data reviewed in this work show that these biomarker classes are consistently altered across diverse malignancies, although the magnitude and direction of change may vary by tumor type and stage [81,106]. Future studies should test whether such signatures identify reproducible patient subgroups and provide information beyond established molecular subtypes and clinical measures [106].

This integrative strategy aligns with contemporary developments in multivariate modeling and systems biology, in which omics-derived data are combined with functional biomarkers to generate predictive algorithms [107,108]. Rather than viewing OS markers as isolated laboratory measurements, their incorporation into precision oncology frameworks positions them as contextual modifiers of molecular information [109]. In this proposed model, genomic data characterize molecular alterations, whereas redox biomarkers could provide complementary information about systemic oxidative status. Their added clinical value remains to be established [110]. Together, these layers may offer a more complete representation of disease heterogeneity and therapeutic responsiveness.

### 5.3. Biomarker-Guided Monitoring Throughout the Cancer Care Continuum

The distinctive translational value of OS biomarkers lies in their dynamic, reversible nature [81]. Unlike fixed genetic alterations, which generally remain stable over the course of disease, redox parameters fluctuate with metabolic activity, inflammatory signaling, treatment exposure, and systemic physiological changes [111]. This temporal sensitivity allows serial measurements to describe changes in systemic oxidative status, although it remains unclear whether these changes reliably track tumor activity or improve clinical decisions. At diagnosis, assessment of lipid peroxidation products, oxidative DNA damage markers, protein oxidation indices, and antioxidant capacity may establish an individualized baseline redox profile. This baseline provides context on systemic oxidative burden before therapy initiation and serves as a reference point for interpreting subsequent changes [81,104].

During anticancer treatment, longitudinal monitoring of OS biomarkers may offer insight into both therapeutic efficacy and host tolerance. Treatment-associated changes in circulating oxidative markers may reflect effects on both tumor and normal tissues. Their ability to distinguish efficacy from toxicity remains unclear [112]. Elevated oxidative damage markers or changes in antioxidant enzyme activity may arise during treatment, but current evidence does not establish that these measurements reliably distinguish treatment toxicity, therapeutic response, or emerging tumor resistance [113,114]. In this context, interpretation should prioritize temporal trends over isolated measurements, as the direction and magnitude of change across successive treatment cycles may be more informative than single-time-point values [81].

In the post-treatment and survivorship phase, continued assessment of redox parameters may help evaluate long-term biological recovery or ongoing stress. Persistent oxidative imbalance after therapy completion could reflect chronic inflammation, residual metabolic dysregulation, or subclinical disease activity [115,116]. Current evidence remains largely observational and does not support incorporating oxidative stress biomarkers into routine post-treatment follow-up protocols. Whether redox profiling adds value to established follow-up strategies remains unclear and requires prospective studies with predefined clinical decisions and outcomes [117,118].

### 5.4. Scientific and Clinical Barriers to Translation

Despite their translational potential, OS biomarkers face several limitations that hinder implementation in routine practice. One fundamental challenge is biological variability. OS levels depend not only on the presence of cancer but also on age, comorbidities, lifestyle, and environmental factors. Distinguishing the neoplastic component from the physiological background is challenging for interpretation [104].

Analytical variability also remains a significant limitation. Differences in assay methods, biological sample types, and preanalytical procedures influence the obtained results. The lack of standardized protocols hinders data comparability and limits the ability to define universal cutoff values [119].

**Figure 2:**
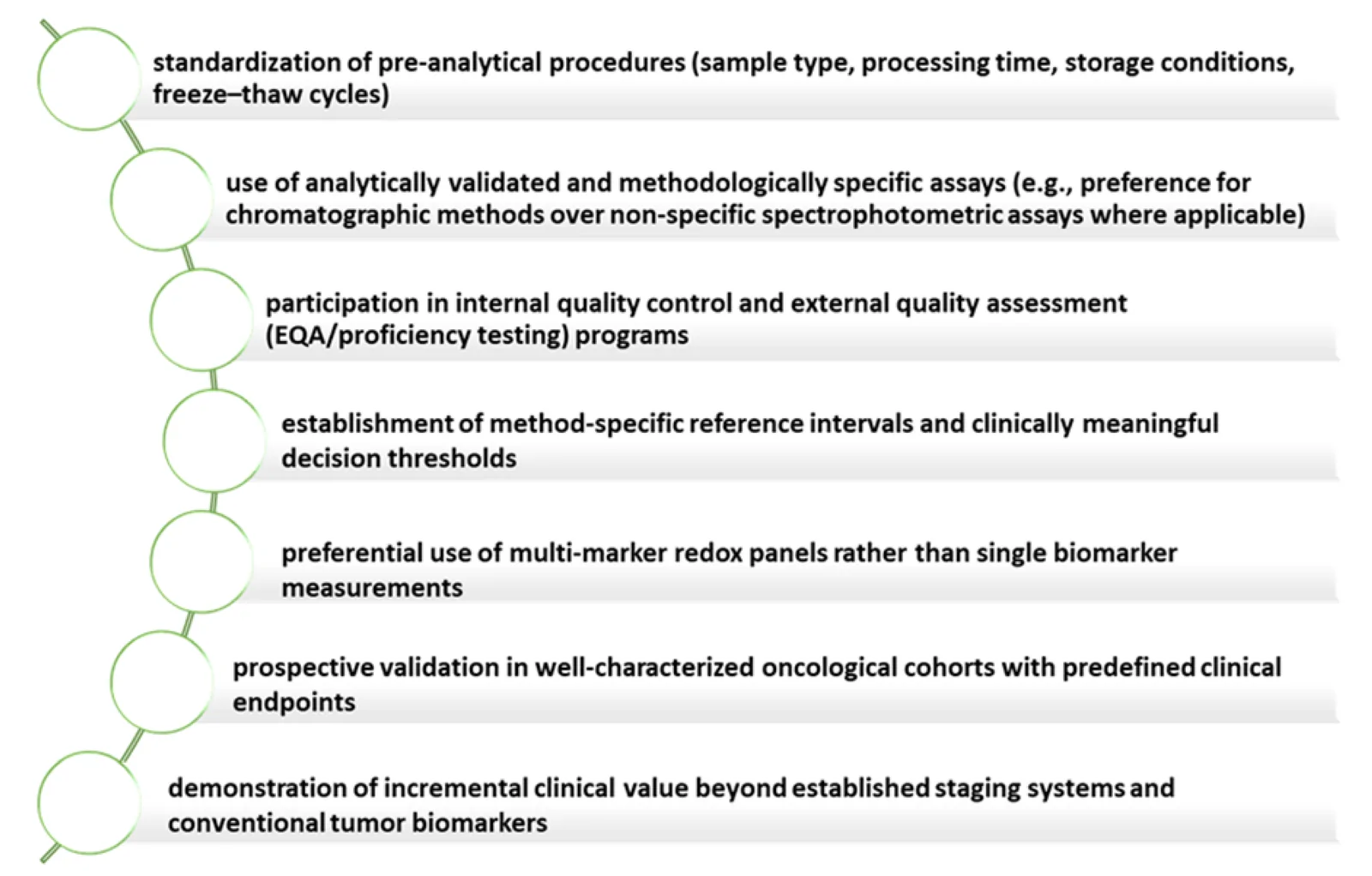
Minimum requirements for clinical adoption of oxidative stress biomarkers.

Another barrier is insufficient prospective validation and a lack of established cutoff points linked to clear clinical decisions. Translating into practice requires demonstrating that redox biomarkers improve prognostic accuracy or treatment effectiveness compared with current standards. In practical terms, translating oxidative stress biomarkers into routine oncology requires meeting several minimal analytical, clinical, and organizational conditions [120]. Figure 2 summarizes these core implementation requirements. Together, they outline the foundational steps necessary to ensure methodological robustness, clinical interpretability, and system-level feasibility of redox biomarker testing.

In summary, OS biomarkers could become a functional component of precision medicine in oncology. They can support risk stratification, therapy personalization, and dynamic patient monitoring. However, full translation into clinical practice requires further method standardization, establishment of clinically useful decision thresholds, and confirmation of added value in prospective studies.

## 6. Organizational and Quality Management Considerations for Implementing Oxidative Stress Biomarkers

### 6.1. Quality Governance and Reliability Requirements for Oxidative Biomarker Testing

Implementing OS biomarker testing in oncology requires governance frameworks that ensure analytical reliability, metrological traceability, and organizational accountability across the full testing pathway. As biomarker assays have diversified across platforms, quality governance has expanded to integrate regulatory requirements, quality management systems, traceability hierarchies, internal and external quality assurance, and formal oversight mechanisms [10,121]. These requirements have assay-specific implications for the biomarkers summarized in Table 2. For example, MDA results from TBARS assays may differ from measurements made with more specific chromatographic methods; 8-oxodG measurements require attention to oxidation during sample preparation and urine normalization; and GSH/GSSG results depend on prompt sample stabilization. Before results from different laboratories can inform the same clinical decision, these sources of variation must be controlled, and methods must be shown to produce comparable results.

Quality Management Systems provide the organizational structure for controlling laboratory processes through documentation, personnel training, risk management, and continuous improvement [122]. International standards such as ISO 15189 [123] and ISO/IEC 17025 [124] formalize requirements for competence, documentation, and management of measurement uncertainty, including the need to define decision rules when issuing statements of conformity [125,126]. Clinical laboratory regulations further specify expectations for quality assurance and quality control, personnel competence, and inspection frameworks irrespective of analytical methodology, which contributes to a governance gap when assays transition from research settings into clinical laboratory practice [127,128].

Laboratory practice frameworks also support governance structures. Good Laboratory Practice standardizes the organization and management of laboratory operations to support reproducibility and reliability [129]. In clinical research contexts, Good Clinical Laboratory Practice integrates principles derived from Good Clinical Practice, GLP, and regulatory requirements into an oversight framework for laboratories generating biomarker data in trials. National regulatory systems, including CLIA, impose additional requirements for validation, quality control, and proficiency testing in clinical laboratories [130,131]. Method development and validation guidance published by professional bodies supports structured analytical evaluation across platforms [128,132].

Reliability governance is reinforced through accreditation and independent performance monitoring. Accreditation provides external verification of laboratory competence and supports auditability of laboratory processes [133]. Documentation requirements issued by organizations such as the College of American Pathologists strengthen traceability of laboratory workflows and reporting procedures [134]. Metrological traceability, defined by an unbroken chain of calibrations linked to recognized references, supports comparability of measurement results across institutions and over time. Calibration hierarchies connecting local standards to primary reference standards maintained by national measurement institutes provide the structural basis for harmonized measurement systems [135]. However, the limited availability of certified reference materials and platform-specific constraints continues to restrict the implementation of fully traceable measurement pathways in several assay domains [136,137].

Quality assurance is operationalized through internal quality control systems and external quality assessment. Internal QA and QC rely on predefined acceptance criteria, stability monitoring, and structured deviation handling, with standard operating procedures supporting workflow consistency [127,138]. External quality assessment and proficiency testing provide independent evaluation through interlaboratory comparison and performance benchmarking, helping detect systematic variability and support harmonization in multi-center settings [139,140]. Integrated oversight frameworks demonstrate how collaborative governance models can coordinate performance monitoring and audit activities across large laboratory networks [129].

Traceability and accountability depend on documentation control and digital infrastructure. Quality manuals and standardized documentation systems support auditability and reproducibility of analytical processes. Laboratory information management systems enable specimen tracking, integration of test outputs with clinical information systems, and structured management of quality records, with compliance features increasingly treated as functional requirements of modern systems [126]. Accountability is maintained through defined organizational roles, oversight mechanisms, and competency management, including training and performance assessment linked to regulated laboratory practice [141].

### 6.2. Standardization and Cross-Institutional Comparability

Differences in analytical platforms, measurement procedures, and reporting practices create structural barriers to cross-institutional comparability of biomarker results. When measurements from the same material cannot be compared across laboratories, the portability of reference intervals and decision thresholds is limited, and integrating results across multi-site clinical and research settings becomes constrained [142,143]. For oxidative stress biomarkers, a reported change in MDA, 8-oxodG, or antioxidant enzyme activity may therefore have a different analytical meaning when laboratories use different assays, sample types, or activity definitions (Table 2). Do not combine these method-dependent results under a common clinical threshold without evidence of comparability.

Within quality governance frameworks, standardization and harmonization represent complementary approaches to comparability. Standardization is achieved when results are calibrated against reference procedures or materials that provide traceable, reproducible measurements across laboratories and over time. Harmonization addresses contexts in which higher-order reference systems are unavailable, aiming to achieve result equivalence within predefined acceptable limits by aligning workflows, calibration approaches, and reporting conventions [142,144]. Many biomarker assays, therefore, operate under harmonization models rather than full metrological standardization [145].

Interlaboratory variability arises throughout the measurement pathway. Pre-analytical variation is associated with collection procedures, processing delays, storage and transport conditions, and freeze–thaw effects, particularly when protocols are not aligned across sites [146,147,148]. Analytical variation reflects differences in assay platforms, reagents and calibrators, instrument configuration, operator technique, and lot-to-lot changes. Post-analytical variation arises from data processing and reporting conventions and from applying method-dependent reference intervals and decision limits [143]. For protein measurands, heterogeneity and proreform complexity can yield method-dependent measurand definitions, limiting direct comparability between assay types and supporting method-specific reference ranges when analytical alignment is not achievable [149].

Technical and organizational interventions support system-level comparability. Standard operating procedures and harmonized workflows reduce procedural dispersion and support reproducibility in multi-laboratory settings [150,151]. Shared reference materials and calibration standards help align measurement scales across instruments and institutions [152]. Automation and standardized platforms can reduce operator-dependent effects and improve interlaboratory agreement, particularly when combined with control of lot-to-lot variability and consistent sample preparation [153]. When reference measurement systems are incomplete, calibration adjustments and harmonized reference intervals provide pragmatic mechanisms to improve agreement between measurement systems [154].

External quality assessment and proficiency testing provide governance mechanisms to monitor comparability and support corrective action. Interlaboratory benchmarking helps identify systematic differences across sites and supports convergence of measurement performance within laboratory networks [155]. Sustaining these activities depends on organizational resources, access to external panels, and integration of quality management functions into routine operations [156].

Comparability also depends on reference-interval governance. Reference interval development depends on analytical methods, reference populations, and statistical procedures, and transferability across institutions is constrained when method-dependent differences persist. Heterogeneity in pediatric and infant reference intervals illustrates how cross-site inconsistency can translate into discordant result flagging even within otherwise aligned laboratory systems [143]. In multi-site contexts, variability in laboratory capacity and operational performance can further affect consistent threshold application and the adoption of standardized testing pathways [157].

Standardization and harmonization activities are embedded within broader regulatory and collaborative infrastructures, including professional guidance, laboratory accreditation frameworks, and cross-institutional networks that disseminate protocols, reference materials, and aligned quality assessment practices [158,159]. Structural barriers remain, including limited availability of commutable reference materials, evolving reporting conventions for high complexity platforms, and the need to align reference intervals and decision thresholds even when analytical procedures converge [152,160].

### 6.3. Organizational Readiness and Implementation Capacity

Implementing OS biomarker testing in routine laboratory practice requires organizational readiness, understood as the capacity of a laboratory and its host healthcare institution to integrate and sustain a new diagnostic process within existing clinical pathways and governance structures [161,162,163]. Readiness extends beyond assay validation and includes infrastructure availability, leadership alignment, workforce capability, and the operational ability to deliver results within predictable turnaround parameters under routine workload conditions [164]. At a system level, implementation capacity reflects whether new testing demands can be absorbed without disrupting existing diagnostic services, including the ability to manage workload, standardize documentation, and maintain performance monitoring after service introduction [165,166].

Implementation frameworks such as CFIR and NASSS provide structured approaches for assessing readiness and anticipating barriers. Evidence across healthcare settings shows that adoption is strongly influenced by workflow compatibility, resource availability, technical dependencies, and trust in diagnostic outputs, all of which determine whether new testing processes can be reliably integrated into routine decision pathways [161,162].

Operational readiness is closely linked to workflow integration across laboratory and clinical environments. Integration with laboratory information systems and electronic health records supports traceability, standardized reporting, and timely access to results. In contrast, limited interoperability or data quality constraints may increase workload and reduce practical usability [167]. Readiness assessments, therefore, require evaluation of documentation pathways, result routing, and compatibility with existing diagnostic algorithms, including the ability to meet clinically appropriate turnaround expectations [168,169].

Turnaround time and workload management are central operational determinants of implementation capacity. New biomarker assays introduce additional tasks across specimen handling, analysis, verification, and reporting, requiring capacity planning, role adjustment, and coordinated workflow design to maintain service efficiency [170,171]. Training, competency assessment, and ongoing performance monitoring are necessary to ensure consistent operation across sites and over time [172,173]. Organizational readiness is also shaped by decisions about centralized or decentralized testing models, each with distinct requirements for infrastructure, workforce capability, and service coordination [160,163].

From a quality governance perspective, implementation capacity depends on coordinated control of workflow design, information system integration, workforce competency, and operational monitoring. These elements determine whether oxidative stress biomarker testing can be delivered consistently and integrated effectively into routine diagnostic practice under sustained service conditions [174].

### 6.4. System-Level Resource Allocation and Policy Implications

Implementing OS in oncology is not only a laboratory decision but a health-system allocation choice, as adoption competes for finite resources and must align clinical benefit, analytical reliability, and service sustainability with broader policy priorities. Across diagnostic domains, translation from technical validation to routine use is frequently delayed by reimbursement uncertainty, infrastructure constraints, fragmented governance, and the need to reconfigure diagnostic pathways across multiple organizational units [175,176,177]. Sustained implementation, therefore, depends on a credible value proposition, organizational capacity, and supportive system conditions, including regulatory frameworks, professional standards, and workflow integration [178].

Financial sustainability remains a central constraint. Administrative and payer pathways can lead to substantial attrition across test requests, authorizations, and reimbursements. At the same time, ongoing operational costs, such as workforce training, quality assurance, and data infrastructure, may exceed initial procurement expenditure [176,179]. In many settings, infrastructure, staffing, and quality system limitations constrain diagnostic capacity, indicating that biomarker implementation may require foundational investment rather than incremental service expansion [180,181]. To maintain system value, adoption strategies increasingly rely on utilization management and iterative technology assessment that incorporates real-world performance, pathway effects, and equity considerations rather than one-time approval decisions [182].

Health technology assessment (HTA) plays a central role in these allocation decisions but remains methodologically challenging for diagnostics. Biomarker evaluation requires models that represent the full causal pathway from analytical performance to clinical decision-making and downstream outcomes [183,184]. Conventional cost-per-QALY approaches may underestimate diagnostic value when benefits arise through pathway modification, reduced uncertainty, or long-term system effects, supporting expanded value frameworks and early HTA approaches that guide evidence generation and prioritize high-impact clinical applications [185,186].

Regulatory–HTA misalignment further shapes implementation. Regulatory approval typically emphasizes analytical validity and product performance. In contrast, HTA and payer decisions require evidence of comparative effectiveness and net clinical benefit, creating duplicate evidence requirements and delaying coverage [187]. Coordinated assessment processes and parallel scientific advice have been proposed to reduce the cumulative evidence burden while maintaining accountability [188]. In oncology, these governance challenges are intensified when biomarker testing defines treatment eligibility, as diagnostic categorization redistributes accountability among regulatory, clinical, and laboratory actors and therefore requires transparent, reviewable classification frameworks [189,190].

Data governance is a further prerequisite for responsible scale-up. Interoperable data systems, secure data-sharing mechanisms, and structured access to real-world data enable monitoring of clinical impact, validation of value claims, and continuous refinement of thresholds and diagnostic pathways [191,192]. Where biomarker outputs inform clinical decision support or AI-enabled processes, explicit accountability structures are required to address transparency, bias, privacy, and liability [193,194].

Overall, responsible integration of oxidative stress biomarkers requires strategic investment in capacity and digital infrastructure, evaluation frameworks that capture system-level value, and policy mechanisms that align reimbursement, regulation, and data governance with routine oncology practice, ensuring that implementation delivers measurable quality improvement rather than additional complexity or unwarranted variation [175,185,186].

## 7. Research Gaps and Future Directions

One of the most important unmet needs is the lack of large, well-designed prospective clinical studies. Large-scale prospective cohort studies with long-term follow-up are necessary to determine whether oxidative stress biomarkers can reliably predict cancer development, disease progression, treatment response, or clinical outcomes across different malignancies [81,100].

A further research priority is to harmonize pre-analytical and analytical procedures and validate clinically meaningful decision thresholds, so that biomarker results can be interpreted reliably across studies and laboratories [195]. Future research should also integrate OS biomarkers into multi-omics frameworks to improve diagnostic and prognostic performance [196]. Such multi-parametric approaches may enhance patient stratification and support precision oncology strategies.

Further investigation is needed to clarify the clinical utility of oxidative biomarkers for monitoring disease dynamics and treatment-related responses. Longitudinal studies evaluating biomarker trajectories before, during, and after anticancer therapy may help determine their potential role in assessing treatment-induced oxidative injury, therapeutic efficacy, and recovery of redox homeostasis.

Finally, future research should consider the feasibility and relevance of implementing OS biomarkers in real-world clinical settings. Although experimental and clinical studies provide encouraging evidence, additional validation in diverse patient populations and routine clinical environments is necessary to confirm their robustness, reproducibility, and clinical interpretability. Establishing clear clinical contexts in which oxidative biomarkers add value beyond existing diagnostic or prognostic tools will be essential for defining their appropriate role in modern oncology.

## 8. Conclusions

This review highlights oxidative stress as a fundamental factor in carcinogenesis and cancer progression, influencing genomic instability, metabolic reprogramming, immune modulation, angiogenesis, and treatment resistance. Studies of lipid peroxidation, protein oxidation, oxidative DNA damage, and antioxidant defenses provide evidence of systemic redox alterations in several malignancies, although biomarker results vary across tumor types, biological matrices, and analytical methods.

Clinically, oxidative stress biomarkers demonstrate reproducible changes in cancer patients and, in selected cases, associations with disease burden and treatment dynamics. However, methodological heterogeneity, a lack of standardized thresholds, and insufficient prospective validation currently limit their routine use. Currently, they are best viewed as complementary elements in multiparametric models rather than as independent diagnostic or prognostic tools.

The direction and magnitude of biomarker changes vary across tumor types, biological matrices, analytical methods, and patient populations, limiting transferability between studies and clinical settings. Clinical adoption therefore requires assay-specific quality control and harmonization, validation in defined patient populations, and evidence that biomarker results improve decisions beyond existing tests. Until these conditions are met, the biomarkers reviewed here remain research tools or markers of clinical association rather than established standalone tests for routine oncology decision-making.

## Figures and Tables

**Table 2:** Analytical and pre-analytical considerations for commonly studied oxidative stress biomarkers.

Marker	Typical Material and Method	Key Pre-Analytical and Analytical Issues	Current Status of Application in Oncology
MDA	Serum/plasma; TBARS, HPLC or LC–MS/MS	TBARS has limited specificity; sample collection, storage, and the assay method are important.	Clinical associations; no approved decision threshold.
4-HNE	Tissue, serum/plasma; immunoassay or chromatography for specific forms	Free 4-HNE and 4-HNE–protein adducts are different measurands; results also depend on sample handling and assay specificity, and should not be treated as interchangeable.	Research-oriented
8-oxodG/8-OHdG	Urine, blood, tissue; LC–MS/MS, HPLC–ECD, or immunoassay	Risk of oxidation during DNA sample preparation; differences between methods and urine normalization.	Clinical associations; no validated decision-making application.
AOPP/protein carbonyls	Serum/plasma; spectrophotometric or immunochemical methods	Results depend on protein composition, sample preparation, and assay chemistry; values obtained with different spectrophotometric or immunochemical methods may not be directly comparable.	Research applications; clinical utility not established
GSH/GSSG	Whole blood, erythrocytes, or tissue; enzymatic or chromatographic methods	Rapid transformation after sampling; strict stabilization conditions required.	Research-oriented
SOD/GPx	Erythrocytes, whole blood, or plasma; activity tests	Results depend on the material fraction, storage conditions, and the definition of activity.	Research-oriented

Abbreviations used: 4-HNE, 4-hydroxy-2-nonenal; 8-oxodG, 8-oxo-7,8-dihydro-2′-deoxyguanosine; 8-OHdG, 8-hydroxy-2′-deoxyguanosine; AOPP, advanced oxidation protein products; GPx, glutathione peroxidase; GSH, reduced glutathione; GSSG, oxidized glutathione; HPLC, high-performance liquid chromatography; HPLC–ECD, high-performance liquid chromatography with electrochemical detection; LC–MS/MS, liquid chromatography–tandem mass spectrometry; MDA, malondialdehyde; SOD, superoxide dismutase; TBARS, thiobarbituric acid-reactive substances.

**Table 3:** Clinical readiness of oxidative stress biomarkers discussed in this review.

Marker	Level of Evidence Identified in this Review	Evidence Needed Before Clinical Use
MDA	Clinical association	Comparable methods, clinical specificity, threshold validation
4-HNE	Exploratory	Standardization of the designated form and prospective validation
8-oxodG/8-OHdG	Clinical association	Validation in the target population and added value compared to current tests
AOPP/protein carbonyls	Clinical association (exploratory), depending on tumor type and endpoint.	Replication and assessment of independence from confounding factors
GSH/GSSG, SOD, GPx	Exploratory	Standardization of the matrix and procedures, and demonstration of clinical utility.

Abbreviations used: 4-HNE, 4-hydroxy-2-nonenal; 8-oxodG, 8-oxo-7,8-dihydro-2′-deoxyguanosine; 8-OHdG, 8-hydroxy-2′-deoxyguanosine; AOPP, advanced oxidation protein products; GPx, glutathione peroxidase; GSH, reduced glutathione; GSSG, oxidized glutathione; MDA, malondialdehyde; SOD, superoxide dismutase. “Exploratory” denotes evidence insufficient to establish a reproducible clinical association for a defined use. “Clinical association” denotes an observed relationship with cancer status or a clinical characteristic, without adequate validation for clinical decision-making. These are qualitative classifications used in this review, not formal regulatory categories. None of the biomarkers listed has been established here as a standalone test for routine oncology decision-making.

## Data Availability

No new data were created or analyzed in this study. Data sharing is not applicable to this article.

## Abbreviations

The following abbreviations are used in this manuscript:

4-HNE	4-hydroxynonenal
8-OHdG	8-hydroxy-2′-deoxyguanosine
8-OHG	8-hydroxyguanosine
8-oxodG	8-oxo-7,8-dihydro-2′-deoxyguanosine
ACSL4	acyl-CoA synthetase long-chain family member 4
AI	artificial intelligence
AOPP	advanced oxidation protein products
ARE	antioxidant response element
AUC	area under the curve
BCR-ABL	breakpoint cluster region–Abelson tyrosine kinase
BMI	body mass index
CAFs	cancer-associated fibroblasts
CAT	catalase
CEA	carcinoembryonic antigen
CFIR	Consolidated Framework for Implementation Research
CLIA	Clinical Laboratory Improvement Amendments
CRC	colorectal cancer
DNA	deoxyribonucleic acid
DNMTs	DNA methyltransferases
ECM	extracellular matrix
ELISA	enzyme-linked immunosorbent assay
EMT	epithelial-to-mesenchymal transition
ERK	extracellular signal-regulated kinase
FH	fumarate hydratase
FRAP	ferric reducing antioxidant power
GLP	Good Laboratory Practice
GPx	glutathione peroxidase
GSH	reduced glutathione
GSH-Px	glutathione peroxidase
GSSG	oxidized glutathione
HER2	human epidermal growth factor receptor 2
HIF-1	hypoxia-inducible factor 1
HPLC	high-performance liquid chromatography
HTA	health technology assessment
IAPs	inhibitors of apoptosis proteins
iNOS	inducible nitric oxide synthase
ISO	International Organization for Standardization
KEAP1	Kelch-like ECH-associated protein 1
LDL	low-density lipoprotein
LPCAT3	lysophosphatidylcholine acyltransferase 3
LPO	lipid peroxidation
MAPK	mitogen-activated protein kinase
MDA	malondialdehyde
MMP	matrix metalloproteinase
MPO	myeloperoxidase
NADPH	nicotinamide adenine dinucleotide phosphate
NF-κB	nuclear factor kappa B
NOX	NADPH oxidase
NRF2	nuclear factor erythroid 2-related factor 2
NSCLC	non-small cell lung cancer
OS	oxidative stress
OSCC	oral squamous cell carcinoma
PBMC	peripheral blood mononuclear cell
PD-1	programmed cell death protein 1
PD-L1	programmed death-ligand 1
PI3K	phosphoinositide 3-kinase
PON1	paraoxonase 1
PTEN	phosphatase and tensin homolog
PUFAs	polyunsaturated fatty acids
QA	quality assurance
QALY	quality-adjusted life year
QC	quality control
RCC	renal cell carcinoma
RNS	reactive nitrogen species
ROS	reactive oxygen species
SDH	succinate dehydrogenase
SIRT3	sirtuin 3
SOD	superoxide dismutase
TAC	total antioxidant capacity
TME	tumor microenvironment
TNM	tumor–node–metastasis
VEGF	vascular endothelial growth factor
XO	xanthine oxidase
